# Microbiome Response to Hot Water Treatment and Potential Synergy With Biological Control on Stored Apples

**DOI:** 10.3389/fmicb.2019.02502

**Published:** 2019-11-06

**Authors:** Birgit Wassermann, Peter Kusstatscher, Gabriele Berg

**Affiliations:** Graz University of Technology, Institute of Environmental Biotechnology, Graz, Austria

**Keywords:** *Malus domestica*, microbiota, postharvest losses, biological control consortium, *Neofabraea sp.*, bull’s eye rot, *Penicillium expansum*, blue mold

## Abstract

Postharvest food decay is one major issue for today’s food loss along the supply chain. Hot water treatment (HWT), a sustainable method to reduce pathogen-induced postharvest fruit decay, has been proven to be effective on a variety of crops. However, the microbiome response to HWT is still unknown, and the role of postharvest microbiota for fruit quality is largely unexplored. To study both, we applied a combined approach of metabarcoding analysis and real time qPCR for microbiome tracking. Overall, HWT was highly effective in reducing rot symptoms on apples under commercial conditions, and induced only slight changes to the fungal microbiota, and insignificantly affected the bacterial community. Pathogen infection, however, significantly decreased the bacterial and fungal diversity, and especially rare taxa were almost eradicated in diseased apples. Here, about 90% of the total fungal community was composed by co-occurring storage pathogens *Neofabraea alba* and *Penicillium expansum.* Additionally, the prokaryote to eukaryote ratio, almost balanced in apples before storage, was shifted to 0.6% bacteria and 99.4% fungi in diseased apples, albeit the total bacterial abundance was stable across all samples. Healthy stored apples shared 18 bacterial and 4 fungal taxa that were not found in diseased apples; therefore, defining a health-related postharvest microbiome. In addition, applying a combined approach of HWT and a biological control consortium consisting of *Pantoea vagans* 14E4, *Bacillus amyloliquefaciens* 14C9 and *Pseudomonas paralactis* 6F3, were proven to be efficient in reducing both postharvest pathogens. Our results provide first insights into the microbiome response to HWT, and suggest a combined treatment with biological control agents.

## Introduction

Food loss is one of the major problems of modern society; about one-third of all produced food is either lost or wasted globally (FAO, 2015a). Especially, the postharvest period plays a crucial role and has a lot of potential for improvements (Kader, 2003; Aulakh and Regmi, 2013). A high proportion of postharvest food loss is induced by postharvest pathogens colonizing and damaging the fruits (Johnston et al., 2002; Morales et al., 2010). Until now, mainly chemical and physical treatments are used to suppress pathogens; microbiome research is expected to bring notable understanding and improvements into future biological applications and treatments (Janisiewicz and Korsten, 2002; Droby and Wisniewski, 2018).

Plants closely interact with their colonizing microorganisms, which are crucial for plant health and growth (Berg, 2009; Berendsen et al., 2012; Vandenkoornhuyse et al., 2015). Microorganisms not only protect the plant before harvest, even after harvest, the shielding effect is prolonged (Droby et al., 2016). Studying plant-microbe interactions, beneficial bacteria and their functions were shown to be substantial for advanced biotechnological agriculture applications (Berg et al., 2017). Even though the development of biocontrol application for postharvest use can be challenging, numerous biocontrol products were developed over the last decades as an alternative to classical synthetic pesticides not only for on-field, but also for postharvest applications (Droby et al., 2016). Additionally, health considerations and potential prohibition of currently used pesticides as well as trends toward a fully biological production increased the demand for highly efficient biological alternatives over the last years (Droby et al., 2009). To increase the efficiency of biological control product also, a combined approach of classical and biological methods was suggested (Porat et al., 2002; Spadaro and Gullino, 2005).

Apple, with worldwide over 83 million tons harvested each year with China, the US, and Poland being the top producers, is one of the major fruit crops worldwide (FAOSTAT, 2017). Apples are stored for several months (up to 12 months) under controlled air conditions (Thompson et al., 2018). Prolonged storage of apples has been investigated around the globe and several technologies have been developed to mitigate pathological and physiological disorders during storage (Janisiewicz and Korsten, 2002; Konopacka and Plocharski, 2004; Thompson et al., 2018). Postharvest fungal infections, however, still cause major shortfalls during storage and along the supply chain (FAO, 2015b). *Penicillium expansum* Link, causing blue mold and the three *Neofabraea* species *N. alba* Jacks, *N. malicorticis* (Jacks) Nannfeld and *N. perennans* Kienholz, being the causal agents of bull’s eye rot, also referred to as gloeosporium rot (Snowdon, 1990) or bitter rot (Corke, 1956) are two of the main postharvest pathogens of apple. Apart from chemical treatments to control postharvest pathogens, hot water treatment (HWT) for 3 min at 50–53°C, a relatively simple method that is used since the 20th century, was shown to be rather effective in reducing pathogen-induced postharvest losses (Fallik et al., 2001; Maxin et al., 2012b, 2014); both bull’s eye rot and blue mold haven been proven to be successfully controlled by HWT (Trierweiler et al., 2003; Maxin et al., 2005). Additionally to the direct killing of fungal spores, the efficiency of HWT is also based on a physiological plant response by inducing transcription and translation of heat shock proteins, where a subset of which comprise pathogenesis-related proteins (Lurie, 1998; Fallik et al., 2001; Pavoncello et al., 2001; Maxin et al., 2012a). However, HWT can cause heat damage to the fruit surface and therefore, additional biocontrol approaches could further improve fruit storability (Spadaro et al., 2004; Schloffer and Linhard, 2016). Recently, combined approaches of HWT with bioactive molecules and biocontrol agents were proven to be efficient in controlling postharvest diseases in apples and other fruits (Conway et al., 2004; Spadaro et al., 2004). Even though these developments were promising, there are still missing links between postharvest diseases on apples and their colonizing microbiota following postharvest treatments including the impact of HWTs on the latter.

The present study provides the first investigation of the apple microbiome changes induced by the currently in-use HWT at an industrial scale. Stored apples that were not subjected to HWT and remaining unaffected by fungal infection and rot development, were investigated, giving some potential insights to postharvest pathogen resistance. Additionally, the indigenous apple microbiota were harnessed for the development of biocontrol agents to combat postharvest pathogens *P. expansum* and *N. malicorticis*. Their additive protective pathogen control effect as well as their applicability in the HWT process was evaluated, providing the first evaluation of a combined process with biological control consortia developed from apple epiphytes. This way, an integrative strategy combining the knowledge of the inherent apple microbiome and its postharvest changes with the development of a novel postharvest treatment was applied.

## Materials and Methods

### Experimental Design and Sample Processing

Organically produced apple fruits (*Malus domestica*) of the cultivar “Topaz” were obtained from the organic storage company Rosenbaum Franz GmbH & Co KG (Pöllau, Austria). Apple samples were taken directly after harvest and after a 6-months storage period. Freshly harvested apples were immediately taken to the laboratory and processed under sterile conditions (in the following named “before storage”). For analyzing impact of HWT on the apple microbiota, 100 apples were stored untreated and 100 apples were subjected to HWT by immersing apples in a 53°C water bath for 3 min. The two groups were stored separately but under the same controlled conditions in the company’s storage chamber for 6 months. Directly after opening storage chambers, fungal infection rate by any fungal pathogen on apples was evaluated. HWT was found to be highly efficient as no disease patterns were observed. Among the 100 apples that were untreated 10% were infected, exhibiting rot diameters of 2.5–4 cm in diameter. A subset of each group, consisting of 10 randomly selected apples, was subjected to amplicon analyses; untreated apples were defined into “untreated healthy” and “untreated diseased.” The apples were transported to the laboratory and processed under sterile conditions. One whole apple for each category and sample (“before storage,” “HWT,” “untreated healthy,” and “untreated diseased”) was cut into smaller pieces and homogenized in a Stomacher laboratory blender (BagMixer, Interscience, St. Nom, France) with 40 ml sterile NaCl (0.85%) solution for 3 min. A total of 4 ml of the solution was centrifuged at 16,000 *g* for 20 min and the pellet stored at −70°C for further DNA extraction. This way 10 biological replicates for each category were produced.

### Microbial DNA Extraction and Metabarcoding Library Construction

The resulting pellets from the previous step were subjected to total microbial DNA extraction using the FastDNA SPIN Kit for Soil (MP Biomedicals, Solon, USA) and a FastPrep Instrument (MP Biomedicals, Illkirch, France) for 30 s at 5.0 m/s. Amplicons were prepared in three technical replicates using the primer pair 515f-926r, specific for bacteria and ITS1f-ITS2r specific for fungi. Sequences of primers are listed in Supplementary Table S1. Peptide nucleic acid (PNA) clamps were added to the PCR mix to block amplification of host plastid and mitochondrial 16S DNA (Lundberg et al., 2013). Amplification of the 16S rRNA gene was performed in a total volume of 20 μl [5 × Taq&Go (MP Biomedicals, Illkirch, France), 1.5 μM PNA mix, 10 μM of each primer, PCR-grade water and 1 μl template DNA] under the following cycling conditions: 95°C for 5 min, 35 cycles of 78°C for 5 s, 55°C for 45 s, 72°C for 90 s, and a final elongation at 72°C for 5 min. PCR for amplifying the fungal ITS region was conducted in 20 μl (5 × Taq&Go, 10 μM of each primer, 25 μM MgCl_2_, PCR-grade water and 2 μl template DNA) using the cycling conditions: 94°C for 5 min, 30 cycles of 94°C for 30 s, 58°C for 35 s, 72°C for 40 s and a final elongation at 72°C for 10 min. A nested PCR step was performed to add barcoded primers (10 μM) in a total volume of 30 μl for both 16S rRNA gene and ITS region: 95°C for 5 min, 15 cycles of 95°C for 30 s, 53°C for 30 s, 72°C for 30 s, and a final elongation at 72°C for 5 min. Three technical replicates, conducted for each sample, were combined and purified by Wizard SV Gel and PCR Clean-Up System (Promega, Madison, WI, USA). DNA concentrations were measured with Nanodrop 2000 (Thermo Scientific, Wilmington, DE, USA) and samples were combined in equimolar concentration. The amplicons were sequenced on a Illumina MiSeq v2 (2 × 250 bp) machine.

### Illumina MiSeq Data Evaluation of 16S rRNA Gene and Its Region and Statistics

After joining forward and reversed paired end reads in QIIME 1.9.1, sequencing data was imported into QIIME 22019.1 and demultiplexed following the QIIME 2 tutorials. The DADA2 algorithm was applied for quality filtering, discarding chimeric sequences and to obtain a feature table [containing sequence variants (SVs)] and representative sequences. Feature classification was performed using a Naïve-Bayes feature classifier trained on the Silva132 release (16S rRNA gene) (Quast et al., 2013) or the UNITE v7.2 release (ITS) (Kõljalg et al., 2013). Sequences of features of interest were further identified on species level using NCBI blast alignment tool. Mitochondria and chloroplast reads were discarded from 16S data. Alpha and Beta diversity was investigated running the core diversity script in QIIME 2 rarefying feature tables to the lowest value of reads present in one sample. Core microbiomes (features present in 50% of the samples) were defined for each sample group and core tables were rejoined to obtain barplots and evaluate taxonomic differences. A taxonomy network was constructed on core genera using Cytoscape version 3.5. (Shannon et al., 2003).

Statistical analysis of metabarcoding data was performed using scripts in QIIME 1.9 as well as QIIME2 2019.1. Alpha diversity was tested using the Kruskal-Wallis test and Beta diversity using Analysis of Similarity (ANOSIM) test. Significant differences (alpha ≤ 0.05) in taxa abundance on genus level were calculated using non-parametric Kruskal-Wallis test and False Discovery Rate (FDR) multiple test correction.

### Quantitative Real-Time PCR

A quantitative real-time PCR (qPCR) was conducted to quantify overall bacterial 16S rRNA and fungal ITS gene copy numbers, as well as postharvest pathogens *P. expansum* and *Neofabraea* spp. For specific quantification of bull’s eye rot-causing *Neofabraea* strains, a primer pair (NeoF, NeoR) was selected that specifically targets the highly conserved β-tubulin gene which was found to amplify the three major pathogens associated with bull’s eye rot (*N. alba, N. malicorticis, N. perennans*), but no other related fungi (Cao et al., 2013). The primer pair (Pexp_patF_F, Pexp_patF_R) for *P. expansum* targeted the *patF* gene (involved in the patulin biosynthesis) and was previously tested for its specificity (Tannous et al., 2015). Primer pairs were used each in 5 pmol/μl concentration and are listed in Supplementary Table S1. All reaction mixes contained 5 μl KAPA CYBR Green, 0.5 μl of each primer, 1 μl template DNA (diluted 1:10 in PCR-grade water), adjusted with PCR-grade water to a final volume of 10 μl. Reaction mix for bacterial amplification was supplemented with 0.15 μl PNA mix to block amplification of host-derived 16S rRNA gene copies. Fluorescence intensities were detected using a Rotor-Gene 6000 real-time rotary analyzer (Corbett Research, Sydney, Australia) with the following cycling conditions: Bacteria: 95°C for 5 min, 45 cycles of 95°C for 20 s, 54°C for 30 s, 72°C for 30 s and a final melt curve of 72 to 95°C. Fungi: 95°C for 5 min, 45 cycles of 95°C for 30 s, 58°C for 35 s, 72°C for 40 s and a final melt curve of 72–95°C. *P. expansum*: 95°C for 5 min, 45 cycles of 95°C for 20 s, 65°C for 15 s, 72°C for 15 s and a final melt curve of 72–95°C. *Neofabraea* sp.: 95°C for 5 min, 45 cycles of 95°C for 20 s, 57°C for 15 s, 72°C for 40 s followed by melt curve of 72–95°C. Amplification efficiency of the target was analyzed with the melting curve (Supplementary Figure S1). Three individual qPCR runs were conducted for each replicate. Intermittently occurring gene copy numbers that were found in negative controls were subtracted from the respective sample. Significant differences (*p* ≤ 0.05) of bacterial and fungal gene copy numbers per apple between the different apple groups were calculated using a pairwise Wilcoxon test (Bonferroni correction) and visualized using ggplot2 in R version 3.5.1.

### Small-Scale Storage Experiments

Small scale experiments were conducted to test the efficacy of potential biocontrol agents with and without combined HWT against infection of the fungal pathogens *P. exopansum* ATCC 7861 (Origin: CBS 325.48) and *N. malicorticis* (Jacks) Nannfeld (Origin: DSMZ 62715), selected as representative for bull’s eye rot-causing fungal pathogens. More than 800 bacterial strains, isolates from apples, were tested for antagonistic properties toward the two pathogens by dual-culture *in vitro* assay on Waksman agar (Berg et al., 2002). Bacterial isolates showing highest antagonistic properties toward both fungi were identified by 16S rRNA gene Sanger sequencing (LGC Genomics, Berlin, Germany) and using the NCBI BLAST alignment tool: *Pantoea vagans* 14E4, *Bacillus amyloliquefaciens* 14C9, and *Pseudomonas paralactis* 6F3. Preliminary tests showed *Pantoea vagans* 14E4 as well as a consortium of the three different bacterial strains on equal proportions to have the best control effect. For *in vivo* tests, 30 apples from the cultivar “Topaz” per treatment and pathogen were rinsed with water and four artificial wounds (1 cm in diameter and depth) were cut with a sterile knife around the radius of the fruits. Each apple was artificially infected with *N. malicorticis* (submerged in a 1.6 × 10^5^ conidia/ml solution) or *P. expansum* (10 μl of a 5 × 10^4^ spores/ml solution) and incubated for 24 h at 20°C. For both, the wound pathogen *P. expansum*, and the lenticel rot causing *N. malicorticis* this way an infection could be induced at the wanted locations. Overnight cultures in nutrient broth (Sifin, Berlin, Germany) of bacterial biocontrol strains were cultivated at 30°C and centrifuged at 5,000 rpm for 15 min. The supernatant was discarded and bacterial pellets were resuspended in sterile sodium chloride solution (0.85%). A consortium on equal proportions of all three biocontrol strains was prepared. Suspensions were diluted to an OD_600_ of 0.2 (approximately, 10^6^ cells/ml). Apples, 24 h after inoculation with the fungal pathogens, were treated either with *P. vagans* 14E4 or the consortium by submerging the apples in the prepared solution for 5 s. HWT groups were previously submerged in 53°C hot water for 3 min and allowed to dry. Negative control samples were stored directly after wounding without pathogen infection and positive control samples were stored after infection with *N. malicorticis* and *P. expansum* without further treatment. Results were evaluated after 3 weeks (*P. expansum*) and 5 weeks (*N. malicorticis*) storage period under controlled conditions at 4°C. Supplementary Figure S2 exemplifies the temporally disease progression of *P. expansum* infection, directly, 1 and 3 weeks after wounding. The diameter of infected areas as well as the length of the cuts was measured and statistical significance tested using a pairwise Wilcoxon test (Bonferroni correction) and visualized using ggplot2 in R version 3.5.1.

## Results

### The Structure of the Core Postharvest Microbiota in Apples

**Figure 1:**
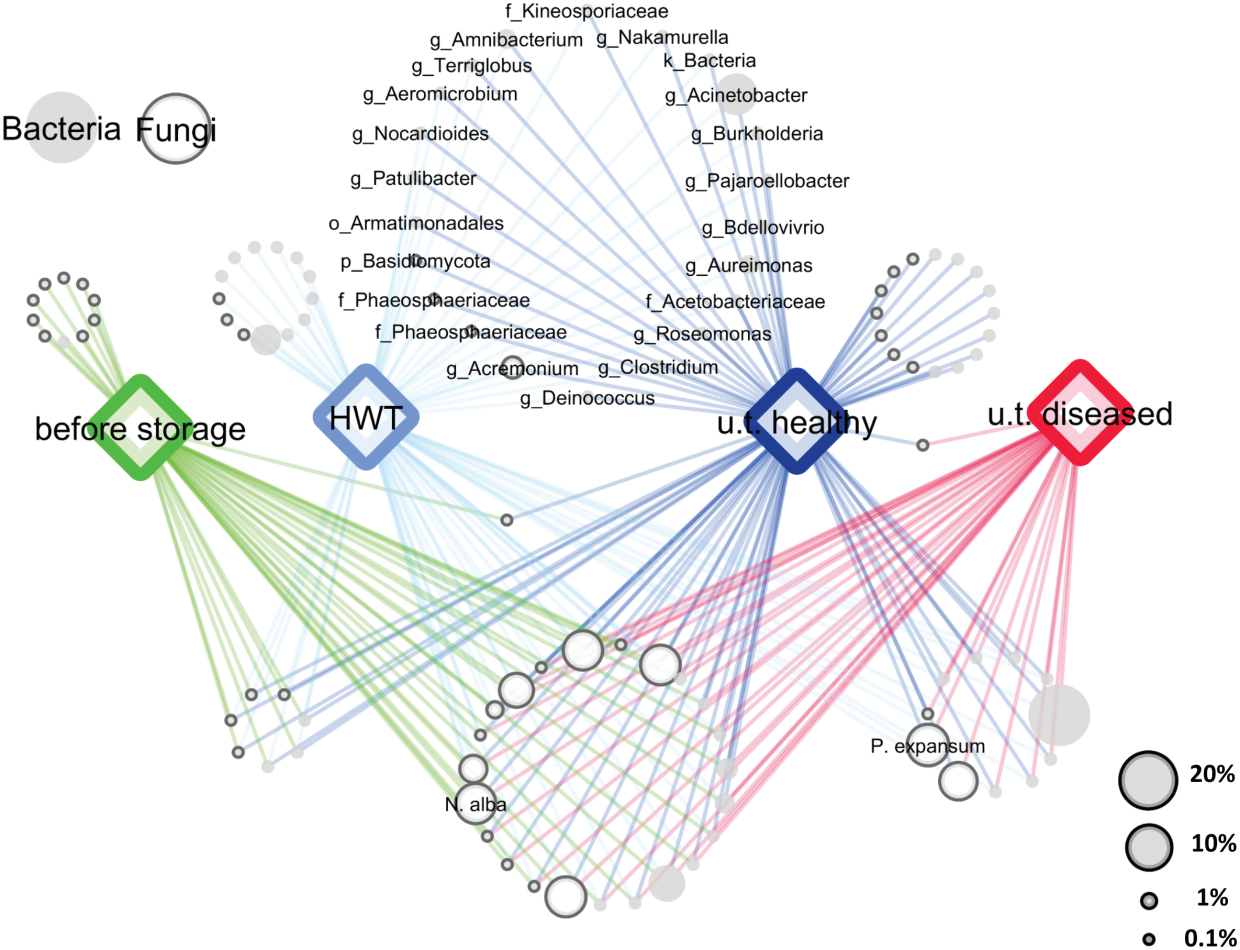
Core and specific microbiota for the four apple groups. Core bacterial and fungal microbiota (taxa occurring in 50% of all replicates) of the four groups “before storage”, “HWT”, “untreated healthy” (u.t. healthy) and “untreated diseased” (u.t. diseased) were combined for network analysis. Node size corresponds to relative abundance in the dataset as described in the legend on the lower right. Node color indicates bacteria (filled light gray) and fungi (outlined dark gray), as shown in the legend on the upper left. Nodes of taxa shared by healthy stored apples, indicating the healthy postharvest microbiota, are labeled where the label prefixes k_, p_, o_, f_ and g_ denote for kingdom, phylum, order, family and genus, respectively. Taxonomy of the two postharvest pathogens *N. alba* and *P. expansum* was assigned on species level using the NCBI BLAST alignment tool.

After quality filtering and removing of chimeric sequences using the DADA2 algorithm and excluding mitochondrial and chloroplast sequences from the 16S rRNA gene fragments, the 16S rRNA and ITS datasets contained 1,071,751 and 880,909 paired reads, respectively. Sequences were assigned to 2,297 bacterial and 613 fungal features and the datasets were rarefied to 1,638 bacterial and 1,319 fungal sequences, according to the sample with the lowest amount of sequences. Core microbiota were defined for each sample group (“before storage,” “HWT,” “untreated healthy,” and “untreated diseased”), by keeping only the features present in 50% of the replicates of the respective group. A 50% cutoff was used to keep enough features for the evaluation of the main features in all groups and to discard rarely occurring features. In total, 205 core bacterial and 89 core fungal features remained that were condensed to 60 and 44 genera, respectively. From those taxa, a network of co-occurring OTUs was constructed to visualize shared taxa and taxa being unique for a specific group (Figure 1). Among 104 bacterial and fungal genera, 23 were shared by all apples, while 22 genera were present in “HWT” and “untreated healthy” apples but absent in all other samples, probably indicating a health-related postharvest microbiome. Additionally, “HWT,” “untreated healthy,” and “before storage” samples hosted 13, 16, and 10 unique taxa, respectively, while no unique taxa were found for “untreated diseased” apples. *N. alba* was present in all apples, including “before storage” samples, whereas *P. expansum* only occurred in stored apples.

### Taxonomic Changes Induced by Storage and Disease

**Figure 2:**
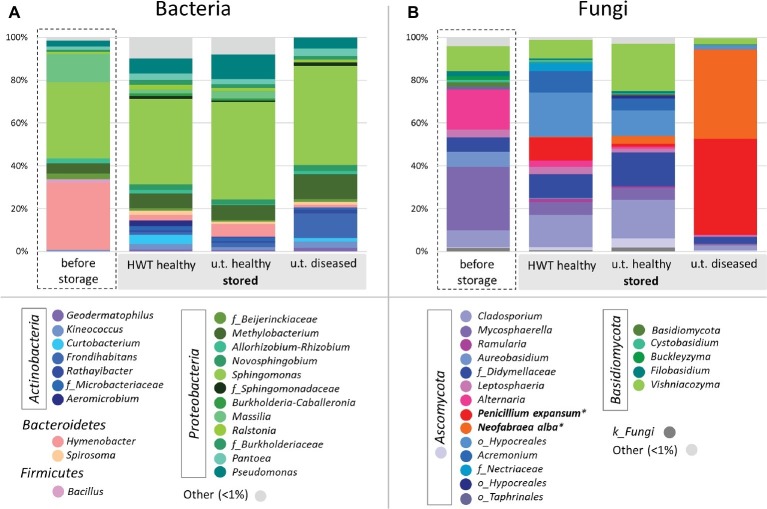
Bacterial and fungal taxonomy of apples investigated. Core microbiomes were defined for taxa occurring in 50% of the replicates in the respective groups. Color-coded bacterial **(A)** and fungal **(B)** taxa are indicated in the bottom legend and are shown on genus level and grouped by phylum. Sequences of storage pathogens highlighted in bold were further identified on species level using NCBI BLAST alignment tool. Taxa occurring with less than 1% are shown as “Other”.

In order to compare taxonomic composition of the four groups, Figure 2 was constructed for the bacterial (Figure 2A) and fungal (Figure 2B) core microbiota of each group on genus level, where genera represented with less than 1% of the number of reads are clustered as “Other.” The microbiota within the four different groups showed great taxonomic variability, especially when apples before storage were compared to stored apples. The bacterial microbiota within all samples was highly dominated by *Proteobacteria,* ranging from 65% in “before storage” samples up to 80% in “untreated healthy” apples. Apples “before storage” had additionally a high abundance of *Bacteroidetes* (32%) compared to the other groups (3–8%), whereas all stored apple samples prevailed in *Actinobacteria* abundance (9–20%) over “before storage” samples (1%). *Sphingomonas* was the most abundant genus in all groups (35–46%). *Hymenobacter* (31%) and *Massilia* (13%) were furthermore highly abundant in apples before storage. *Pseudomonas* (7–11%) and *Methylobacterium* (7%) were abundant in healthy apples after storage, whereas diseased apples after storage showed high abundances of *Methylobacterium* (12%) and *Frondihabitans* (11%) (Figure 2A). In total, the core microbiota of the four groups “before storage”, “HWT”, “untreated healthy” and “untreated diseased” contained 15, 50, 49, and 18 bacterial genera, respectively.

The fungal microbiota was dominated by *Ascomycota,* ranging from 72% in “untreated healthy” samples up to 97% in “untreated diseased” apples. *Basidiomycota* were more abundant in healthy apples before (19%) and after (11–26%) storage, compared to “untreated diseased” apples (3.5%). On genus level, *Mycosphaerella* dominated “before storage” samples (30%), followed by *Alternaria* (19%), *Vishniacozyma* (12%), *Cladosporium* (8%), and *Aureobasidium* (7%). Stored “HWT” samples were dominated by a not further assigned taxon of *Hypocreales* (20%), followed by *Cladosporium* (15%), *P. expansum* (11%), *Acremonium,* and *Didymellaceae* sp. (each 10%) and *Vishniacozyma* (9%). Almost the same fungal genera were highly abundant in stored “untreated healthy” samples, with *Vishniacozyma* (21%) being the main representative, except *P. expansum* featuring only 1% abundance. Stored “untreated diseased” apples were almost exclusively composed of the two postharvest pathogens *P. expansum* (45%) and *N. alba* (42%) (Figure 2B). Both fungi were present in “HWT” and “untreated healthy” apples, although with less relative abundance. “Before storage” apples contained 0.1% *N. alba*, while *P. expansum* was absent. The samples “before storage”, “HWT”, “untreated healthy” and “untreated diseased” contained 28, 27, 33, and 18 fungal core genera, respectively.

### Diversity Changes Induced by Storage and Disease

**Figure 3:**
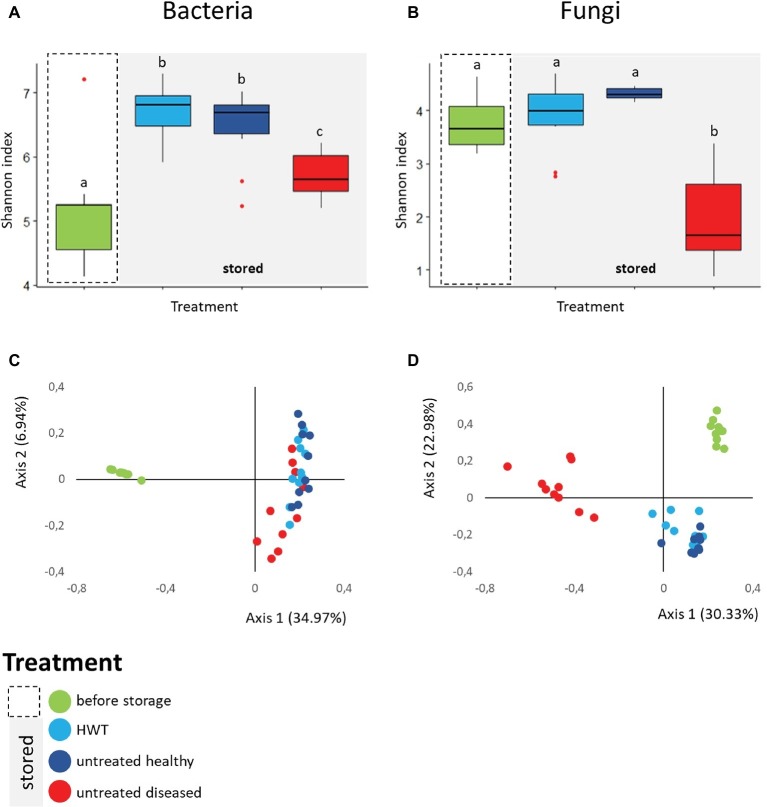
Alpha- and Beta-diversity analyses on apple-associated bacterial and fungal structure. Box-and-Whiskers-plots visualize Shannon diversity index of the four different apple groups for bacteria **(A)** and fungi **(B)**. Significant differences (*p* ≤ 0.05) were assessed by Kruskal Wallis test and are indicated by different lower case letters. Community clustering of bacterial **(C)** and fungal **(D)** composition of the samples is indicated by color-coded two dimensional Bray Curtis PCoA plots. Color code for the differentially treated apple samples is explained in the legend on the bottom left. Significant differences in bacterial and fungal composition was tested using ANOSIM pairwise test and can be looked up in Table 1.

The bacterial and fungal diversity within the apple samples was assessed by Shannon diversity index. Apples from the category “before storage” showed significantly the lowest bacterial diversity (H′ = 5.19 ± 0.8), followed by stored apples from the category “untreated diseased” (H′ = 5.72 ± 0.3). Both were significantly less diverse than stored “untreated healthy” (H′ = 6.46 ± 0.6) and “HWT” samples featuring highest bacterial diversity (H′ = 6.68 ± 0.4) (Figure 3A). Fungal diversity was highly decreased in stored “untreated diseased” apples (H′ = 1.93 ± 0.8), being significantly lower compared to all healthy apples: “before storage”: H′ = 3.77 ± 0.5, “HWT”: H′ = 3.87 ± 0.6 and “untreated healthy”: H′ = 4.31 ± 0.1 (Figure 3B).

Beta diversity analyses, applied on the whole bacterial and fungal dataset and based on Bray Curtis distance matrix, indicated clear clustering between apples before and after storage in all cases (Figures 3C,D). Statistical significance in bacterial composition, assessed *via* pairwise ANOSIM (Table 1), revealed significant differences between all groups, except for the comparison of “HWT” and “untreated healthy” samples. Highest variability was found when “before storage” samples were compared to the remaining groups. The fungal composition was significantly different between all four groups, while difference between “HWT” and “untreated healthy” samples was lowest.

**Table 1:** Pairwise ANOSIM results calculating significant differences in bacterial and fungal composition associated with differentially treated apple groups.

		Bacteria		Fungi	
Group 1	Group 2	*R*	*p*	*R*	*p*
HWT	Untreated diseased	0.21	0.002	0.79	0.001
HWT	Untreated healthy	0.06	0.136	0.41	0.001
HWT	Before storage	1.00	0.001	0.95	0.001
Untreated diseased	Untreated healthy	0.26	0.001	0.81	0.001
Untreated diseased	Before storage	1.00	0.001	0.85	0.001
Untreated healthy	Before storage	1.00	0.001	1.00	0.001

In order to identify bacterial and fungal taxa that potentially contribute to pathogen resistance in “untreated healthy” apples, significant differences in taxa abundance between “untreated healthy” and “untreated diseased” samples were calculated (Supplementary Table S2). A total of 42 bacterial and 28 fungal taxa were found significantly higher in “untreated healthy” apples as well as 2 fungal taxa (*P. expansum* and *N. alba*) being significantly increased in “untreated diseased” apples. Higher numbers of taxa in “untreated healthy” apples were found for e.g., *Sphingomonas*, *Pseudomonas,* and *Methylobacterium* as well as *Vishniacozyma*, *Cladosporium,* and *Acremonium*.

Additionally, the impact of HWT on the apple postharvest microbiota was evaluated as well, by calculating significant differences in taxa abundance between “HWT” and “untreated healthy” apples (Supplementary Table S3). A total of 25 bacterial and 22 fungal genera were found to be significantly different abundant between the two groups. Significantly increased in “HWT” were, e.g., *Hymenobacter*, *Rathayibacter* as well as *Filobasidium*; increased in “untreated healthy” were, e.g., *Curtobacterium*, *Rhodococcus* as well as *Penicillium* and *Alternaria*. However, as previous stated, the overall bacterial microbiome and diversity was not significantly different between the two groups only the fungal microbial composition was slightly changed.

### Quantification of Bacteria, Fungi, *P. expansum,* and *Neofabraea* Sp. During Storage and Disease

**Figure 4:**
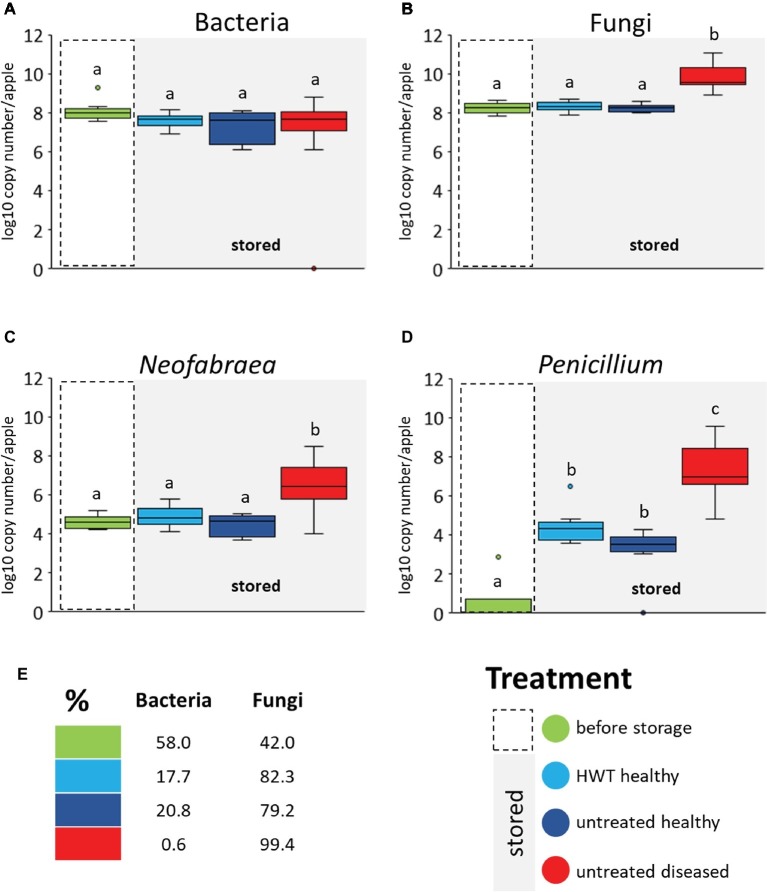
Microbial gene copy numbers in apple groups determined by qPCR. Values are given by primers targeting bacterial 16S rRNA genes **(A)**, fungal ITS region **(B)** and genes of *N. alba* **(C)** and *P. expansum* **(D)**. Gene copy numbers are calculated per apple used for the microbiome analysis. Significant differences (*p* ≤ 0.05) were assessed by Wilcoxon test (Bonferroni correction) and are indicated by different lower case letters. The prokaryote to eukaryote ratio within the total microbial gene copies detected in apples of the respective groups is shown **(E)**. Color code for apple groups is depicted in the legend on the bottom right.

A real time PCR was performed to quantify total bacterial 16S rRNA and fungal ITS gene copy numbers. Bull’s eye rot-causing *Neofabraea* strains and *P. expansum* were specifically quantified as well (Figure 4). No significant differences in 16S rRNA gene copy abundance was observed between the four different apple groups; neither between apple “before storage” and all stored apples, nor within the stored groups (Figure 4A). Pathogen infection as well as HWT did accordingly not affect the bacterial abundance in apples. Regarding the total fungal ITS genes we found significantly higher abundances within “untreated diseased” apples compared to all other groups (Figure 4B), due to significant increase of both storage pathogens *Neofabraea* and *P. expansum* (Figures 4C,D, respectively). *Neofabraea* was already present in “before storage” apples in similar abundances as in “HWT” and “untreated healthy” apples while *P. expansum* was almost absent in apples “before storage.” Overall, fungi were found to proliferate more efficiently compared to bacteria in stored apples, as showed *via* calculating the prokaryote to eukaryote ratio (Figure 4E). Whereas the ratio was almost balanced in apples before storage (58% bacteria and 42% fungi), fungal genes increased up to the two-fold in stored, healthy apples. A dramatic increase of fungal genes was however observed within stored, diseased apples; 99.4% of total microbial genes detected were fungal.

### Efficiency of Hot Water Treatment and Biological Control Application Against Postharvest Diseases Determined in Small-Scale Storage Experiments

**Figure 5:**
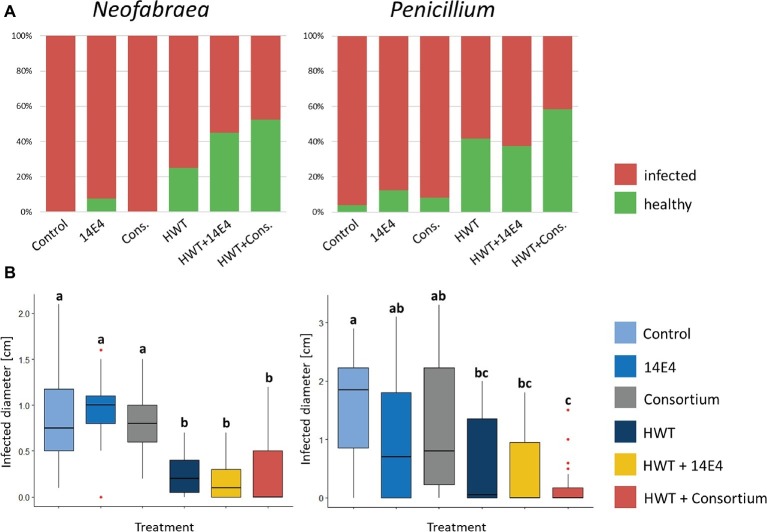
Fraction of infected apples after storage **(A)** and analysis of infected diameter **(B).** Apples were treated with fungal spores or conidia as well as bacterial strain *P. vagans* 14E4, a bacterial consortium and/or HWT. **(A)** Contrasts the total number of infected to healthy apples after treatment and **(B)** statistically evaluates the efficiency of the treatment based on the infection diameter. Statistical differences between differentially treated apple samples was assessed by Wilcoxon test (Bonferroni correction) and are indicated by lower case letters. Control samples were only inoculated with fungal spores and stored.

The efficacy of potential biocontrol strains (*P. vagans* 14E4, *B. amyloliquefaciens* 14C9 and *P. paralactis* 6F3) identified using antagonistic screening methods was tested in small-scale storage experiments with or without combined HWT against *N. malicorticis* and *P. expansum*. *P. vagans* E14 was applied as single agent as well as combined with the other potential biocontrol strains in form of a consortium. Negative control apples that were wounded artificially but not infected with fungal pathogens appeared to be unaffected after two as well as after 5 weeks of storage. Positive control apples that were inoculated with the fungal pathogens and untreated showed 100% infection rate for *N. malicorticis* and 96% for *P. expansum* (Figure 5A). Treatment using biocontrol strains slightly decreased infection rates, however, still up to 88% of apples were infected. HWT reduced infection rates of *N. malicorticis* and *P. expansum* to 58 and 75%, respectively. Overall, combining HWT and the biocontrol consortium reduced the total infection rates the most (up to 42%). Similar results were shown when the infection diameter was measured (Figure 5B). Here, no significant differences in infection diameter were found between positive control samples and apples treated with biocontrol strains that were not subjected to HWT. In contrast, HWT approved to be efficient in reducing pathogen infection rates, while the combined treatment of HWT and potential biocontrol strains resulted in even less infection.

## Discussion

The present study is the first to provide deeper insights into the taxonomic, diversity and abundance changes induced by currently in-use HWT at industrial scale. The efficacy of HWT in reducing postharvest pathogens was demonstrated under commercial storage condition. The induced microbial shifts were observed by metabarcoding analysis and microbial quantification *via* qPCR. Small-scale storage experiments furthermore suggest the combination of highly effective HWT and a biological control consortium to be an alternative approach to prevent postharvest loss previously damaged apples.

HWT under commercial storage conditions was proven to be highly efficient as during long-term storage for 6 months, not a single fruit among 100 HW-treated apples was decayed. Among untreated and stored apples, 10% were infected and showed postharvest disease. We studied the induced changes in the microbiome comparing “HWT” and “untreated healthy” apples. The difference between the two groups was not statistically significant for bacteria on any level measured; Alpha and Beta diversity matrixes, as well as gene quantification revealed no significant differences between the two groups. The fungal composition was, however, slightly influenced. Accordingly, we hypothesize that the apple is protected by the previously suggested HWT-initiated transcription and translation of heat-shock proteins in the plant, where a subset of which comprise pathogenesis-related proteins (Fallik et al., 2001; Pavoncello et al., 2001; Maxin et al., 2014). The HWT as well as the plant response affects the present bacteria to a lesser extent than the fungi. However, still few bacterial and fungal taxa were found to be significantly different abundant between HW-treated and untreated healthy apples, which are therefore suggested to be directly affected by HWT. Whether this microbiota is heat-sensitive or diminished by HWT-induced plant response remains, however, unclear. Among others, also *Penicillium* was significantly reduced in HW-treated apples, which could also be due to the heat sensitivity of fungal spores and structures (Maxin et al., 2012a).

Overall, healthy apples (HWT or untreated) showed a distinct microbiome compared to diseased apples. A total of 18 bacterial and 4 fungal taxa were shared between HW-treated and untreated but healthy apples, while being absent in diseased apples. Explicitly selecting taxa from the healthy postharvest microbiome might provide promising opportunities for future applications to reduce postharvest decay of apples and other fruits.

The impact of pathogen infection on the bacterial and especially on the fungal microbiota of stored apples was severe. Microbial diversity was significantly reduced and the composition was clearly shifted. Even though each replicate consisted of one whole apple fruit and the infection observed was just 2.5 cm in diameter, almost 90% of all fungal sequences detected in diseased apples were composed by co-occurring *N. alba* (42% rel.) and *P. expansum* (45% rel.) and especially the low abundant taxa were almost outcompeted during pathogen infection.

Observing apples before storage, the ratio between bacteria and fungi was almost balanced (58 to 42% for bacteria and fungi, respectively). The ratio shifted toward 20% bacteria and 80% fungi in stored but healthy apples (both HW-treated and untreated samples) and climaxed in 99.4% fungal genes, out of all microbial genes detected, in diseased apples. This percentage was almost exclusively covered by pathogenic *Neofabrea* species and *P. expansum* as detected *via* specific gene quantification, coinciding significantly with the observations in microbiota taxonomy. Even though the infected spots on diseased apples reached a maximum of only 4 cm in diameter on one apple, this emphasizes even more the fast impact of pathogen infestation on the overall microbial composition. The results of this study suggest that the two pathogens are highly co-occurring; moreover, a mutualistic effect is suggested. Outbreaks of pathogenic *Neofabraea* species, known to infect the apple fruit already in the field (Snowdon, 1990), most likely facilitate infestation of rapidly proliferating *P. expansum,* which attacks the fruit through damaged tissues and wounds during storage (Amiri and Bompeix, 2005). After a 6-months storage period this results in a disease outbreak induced by both pathogens to an equal extent. For a significant reduction of *P. expansum* in stored fruits, prevention of *Neofabraea* infection might therefore be essential. The infectious cycles of the two pathogens was confirmed in the present study as well, as *N. alba* was detected already in apples before storage, whereas *P. expansum* was present only in apples stored for 6 months.

Overall, among stored apples, HWT and pathogen infection influenced the bacterial community to a lesser extent than the fungal. Surprisingly, the greatest effect on the bacterial microbiota was mediated by long-term storage. Apples before storage exhibited significantly lower bacterial diversity compared to all stored samples, including diseased apples. The bacterial microbiota was furthermore significantly shifted during storage, whereas bacterial abundance was unchanged across all samples investigated. Storage, therefore, seems to exhibit an even higher effect on the bacterial microbiota than pathogen infestation, whereas the opposite was observed for the fungal community. During storage significant shifts in fungal composition and slight, but not statistically significant increase in diversity was observed. Especially the bacterial genera *Hymenobacter* and *Massilia* and the fungi *Mycosphaerella*, *Alternaria* and *Aureobasidium,* featuring high abundances in apples before storage, were significantly reduced after the 6-months storage period; probably due to cold-sensitivity of those taxa.

Although HWT was shown to be highly efficient in this experiment, it was reported to cause heat damage to a fraction of the stored apples (Schloffer and Linhard, 2016). To prevent postharvest disease in those heat damaged as well as mechanically damaged fruits, biological control was previously suggested (Spadaro et al., 2004). Small-scale experiments demonstrated a significant reduction of symptoms caused by postharvest pathogens *N. malicorticis* and *P. expansum* when fruits were subjected to HWT with or without additional application of a biological control consortium, while the latter even enhanced the efficacy of the treatment. The efficiency was equally pronounced against both pathogens as determined by counting infected apples and measuring diameters of infection on apples artificially wounded and infected with the pathogens. The combined method of HWT and biological control consortium, previously isolated from apples, reduced infection rates up to 42%. Our experiment showed that the fungistatic effect was stable for at least 5 weeks as we evaluated fruit decay after 3 weeks for *P. expansum* and after 5 weeks for *N. malicorticis.* Efficacy of combined methods of HWT and biological control has already been proven successful for apple (Conway et al., 2004; Spadaro et al., 2004), citrus fruits (Porat et al., 2002; Obagwu and Korsten, 2003), pear (Zhang et al., 2008), strawberry (Wszelaki, 2003), mandarin fruit (Hong et al., 2014) and tomato (Zong et al., 2010). However, the present study was the first to test microbial consortia in combination with HWT. Nevertheless, the efficacy of the combined method needs however to be confirmed on industrial scale and with naturally infected fruit.

Until now, only few studies have assessed the microbial dynamics during storage using metagenomic technologies. Investigations on the oomycete and fungal community of sugar beets infection by storage soft rot showed that the susceptibility to storage pathogens was rather conditioned by the cultivar than by the oomycete and fungal community present. Accordingly, plant-inherent but unspecific resistance mechanism was suggested to decrease the spread of pathogens, but without preventing the infection (Liebe et al., 2016). However, the bacterial community, which was not investigated in this study, could potentially contribute to disease expression as well. The dynamic changes of the endophytic bacterial community associated with potato tubers in response to bacterial storage pathogens was investigated by Kõiv et al. (2015). Here, pathogenesis is assumed to be initiated by the pathogen but complex contributions from the endophytic community are significantly involved. A crucial impact of endophytic bacteria and fungi on the development of postharvest stem-end rots was also observed for mango fruits (Diskin et al., 2017). In summary, and with reference to the present results, the severity of postharvest infestations may be rather mediated by the interactions of specific members of the total community than by one specific pathogen. High microbial diversity in plants was already described to determine abundance of pathogens (Berg et al., 2017).

## Conclusion

The indigenous apple microbiome is important for health within the postharvest period and during storage. A healthy apple microbiome is characterized by high bacterial and fungal diversity and evenness, a balanced ratio between both groups and several health indicators, while diseased apples show dysbiosis, diversity loss and dominant fungal pathogens. HWT-induced plant response diminished pathogen infection at industrial scale, and showed an impact on the fungal composition. We suggest that the apple fruit is protected by either HWT or the inherent microbiome; however, presumable it is the combination of both, mediating disease resistance. Small-scale storage experiments applying HWT together with biological control agents provide further confirmation of the considerable potential of combining methods into one control strategy to reduce postharvest decay of apples. Moreover, harnessing the indigenous microbiota of fruits for a biological control approach is a promising and sustainable future strategy to prevent postharvest decay of fresh and stored produce.

## Data Availability Statement

The raw sequence files supporting the findings of this article are available from the European Nucleotide Archive (ENA) at study Accession Number PRJEB33672.

## Author Contributions

BW and PK performed the experiments, analyzed data, and wrote the manuscript. GB designed the study, discussed results, and wrote the manuscript. All authors read and approved the final manuscript.

### Conflict of Interest

The authors declare that the research was conducted in the absence of any commercial or financial relationships that could be construed as a potential conflict of interest.

## References

[ref1] Amiri A. Bompeix G. (2005). Diversity and population dynamics of Penicillium spp. on apples in pre- and postharvest environments: consequences for decay development. Plant Pathol. 54, 74–81. 10.1111/j.1365-3059.2005.01112.x

[ref2] Aulakh J. Regmi A. (2013). Post-harvest food losses estimation-development of consistent methodology. Rome: FAO.

[ref3] Berendsen R. L. Pieterse C. M. J. Bakker P. A. H. M. (2012). The rhizosphere microbiome and plant health. Trends Plant Sci. 17, 478–486. 10.1016/j.tplants.2012.04.001, PMID: 22564542

[ref4] Berg G. (2009). Plant–microbe interactions promoting plant growth and health: perspectives for controlled use of microorganisms in agriculture. Appl. Microbiol. Biotechnol. 84, 11–18. 10.1007/s00253-009-2092-7, PMID: 19568745

[ref5] Berg G. Köberl M. Rybakova D. Müller H. Grosch R. Smalla K. (2017). Plant microbial diversity is suggested as the key to future biocontrol and health trends. FEMS Microbiol. Ecol. 93. 10.1093/femsec/fix050, PMID: 28430944

[ref6] Berg G. Nicotte R. Anette S. Leo E. Angela Z. Korndia S. (2002). Plant dependent genotypic and phenotypic diversity of antagonistic rhizobacteria isolated from different verticillium host plants. Appl. Environ. Microbiol. 68, 3328–3338. 10.1128/AEM.68.7.3328-3338.2002, PMID: 12089011 PMC126805

[ref7] Cao D. Li X. Cao J. Wang W. (2013). PCR detection of the three Neofabraea pathogenic species responsible for apple Bull’s eye rot. Adv. Microbiol. 3, 61–64. 10.4236/aim.2013.31009

[ref8] Conway W. S. Leverentz B. Janisiewicz W. J. Blodgett A. B. Saftner R. A. Camp M. J. (2004). Integrating heat treatment, biocontrol and sodium bicarbonate to reduce postharvest decay of apple caused by *Colletotrichum acutatum* and *Penicillium expansum*. Postharvest Biol. Technol. 34, 11–20. 10.1016/j.postharvbio.2004.05.011

[ref9] Corke A. T. K. (1956). Bitter rot of apples: II. Seasonal variations in the development and sporulation of cankers of Gloeosporium Spp. inoculated into apple branches. J. Hortic. Sci. 31, 272–283. 10.1080/00221589.1956.11513877

[ref10] Diskin S. Feygenberg O. Maurer D. Droby S. Prusky D. Alkan N. (2017). Microbiome alterations are correlated with occurrence of postharvest stem-end rot in mango fruit. Phytobiomes J. 1, 117–127. 10.1094/PBIOMES-05-17-0022-R

[ref11] Droby S. Wisniewski M. (2018). The fruit microbiome: a new frontier for postharvest biocontrol and postharvest biology. Postharvest Biol. Technol. 140, 107–112. 10.1016/j.postharvbio.2018.03.004

[ref12] Droby S. Wisniewski M. Macarisin D. Wilson C. (2009). Twenty years of postharvest biocontrol research: is it time for a new paradigm? Postharvest Biol. Technol. 52, 137–145. 10.1016/j.postharvbio.2008.11.009

[ref13] Droby S. Wisniewski M. Teixidó N. Spadaro D. Jijakli M. H. (2016). The science, development, and commercialization of postharvest biocontrol products. Postharvest Biol. Technol. 122, 22–29. 10.1016/j.postharvbio.2016.04.006

[ref14] Fallik E. Tuvia-Alkalai S. Feng X. Lurie S. (2001). Ripening characterisation and decay development of stored apples after a short pre-storage hot water rinsing and brushing. Innov. Food Sci. Emerg. Technol. 2, 127–132. 10.1016/S1466-8564(01)00032-7

[ref15] FAO (2015a). Food loss and food waste. Rome, Italy: Food and Agriculture Organisation Available at: http://www.fao.org/food-loss-and-food-waste/en/ (Accessed May 5, 2019).

[ref16] FAO (2015b). Post-harvest losses along value and supply chains in the Pacific Island countries. Vol. 1. Rome, Italy: Food and Agriculture Organisation, 1–5.

[ref17] FAOSTAT (2017). Food and agriculture Organization of the United Nations. Rome, Italy: Food and Agriculture Organisation Available at: http://www.fao.org/faostat/en/#data/QC (Accessed May 5, 2019).

[ref18] Hong P. Hao W. Luo J. Chen S. Hu M. Zhong G. (2014). Combination of hot water, *Bacillus amyloliquefaciens* HF-01 and sodium bicarbonate treatments to control postharvest decay of mandarin fruit. Postharvest Biol. Technol. 88, 96–102. 10.1016/j.postharvbio.2013.10.004

[ref19] Janisiewicz W. J. Korsten L. (2002). Biological control of postharvest diseases of fruits. Annu. Rev. Phytopathol. 40, 411–441. 10.1146/annurev.phyto.40.120401.130158, PMID: 12147766

[ref20] Johnston J. W. Hewett E. W. Hertog M. L. A. T. M. (2002). Postharvest softening of apple (*Malus domestica*) fruit: a review. New Zeal. J. Crop Hortic. Sci. 30, 145–160. 10.1080/01140671.2002.9514210

[ref21] Kader A. A. (2003). A perspective on postharvest horticulture (1978-2003). HortScience 38, 1004–1008. 10.21273/HORTSCI.38.5.1004

[ref22] Kõiv V. Roosaare M. Vedler E. Ann Kivistik P. Toppi K. Schryer D. W.. (2015). Microbial population dynamics in response to Pectobacterium atrosepticum infection in potato tubers. Sci. Rep. 5:11606. 10.1038/srep11606, PMID: 26118792 PMC4484245

[ref23] Kõljalg U. Nilsson R. H. Abarenkov K. Tedersoo L. Taylor A. F. S. Bahram M.. (2013). Towards a unified paradigm for sequence-based identification of fungi. Mol. Ecol. 22, 5271–5277. 10.1111/mec.12481, PMID: 24112409

[ref24] Konopacka D. Plocharski W. J. (2004). Effect of storage conditions on the relationship between apple firmness and texture acceptability. Postharvest Biol. Technol. 32, 205–211. 10.1016/j.postharvbio.2003.11.012

[ref25] Liebe S. Wibberg D. Winkler A. Pühler A. Schlüter A. Varrelmann M. (2016). Taxonomic analysis of the microbial community in stored sugar beets using high-throughput sequencing of different marker genes. FEMS Microbiol. Ecol. 92:fiw004. 10.1093/femsec/fiw004, PMID: 26738557

[ref26] Lundberg D. S. Yourstone S. Mieczkowski P. Jones C. D. Dangl J. L. (2013). Practical innovations for high-throughput amplicon sequencing. Nat. Methods 10, 999–1002. 10.1038/nmeth.2634, PMID: 23995388

[ref27] Lurie S. (1998). Postharvest heat treatments. Postharvest Biol. Technol. 14, 257–269. 10.1016/S0925-5214(98)00045-3

[ref28] Maxin P. Huyskens-Keil S. Klopp K. Ebert G. (2005). Control of postharvest decay in organic grown apples by hot water treatment. Acta Hortic. 682, 2153–2158. 10.17660/ActaHortic.2005.682.294

[ref29] Maxin P. Weber R. W. S. Lindhard Pedersen H. Williams M. (2012a). Hot-water dipping of apples to control *Penicillium expansum*, *Neonectria galligena* and *Botrytis cinerea*: effects of temperature on spore germination and fruit rots. Eur. J. Hortic. Sci. 77, 1–9.

[ref30] Maxin P. Weber R. W. S. Pedersen H. L. Williams M. (2012b). Control of a wide range of storage rots in naturally infected apples by hot-water dipping and rinsing. Postharvest Biol. Technol. 70, 25–31. 10.1016/j.postharvbio.2012.04.001

[ref31] Maxin P. Williams M. Weber R. W. S. (2014). Control of fungal storage rots of apples by hot-water treatments: a northern European perspective. Erwerbs-obstbau 56, 25–34. 10.1007/s10341-014-0200-z

[ref32] Morales H. Marín S. Ramos A. J. Sanchis V. (2010). Influence of post-harvest technologies applied during cold storage of apples in *Penicillium expansum* growth and patulin accumulation: a review. Food Control 21, 953–962. 10.1016/j.foodcont.2009.12.016

[ref33] Obagwu J. Korsten L. (2003). Integrated control of citrus green and blue molds using *Bacillus subtilis* in combination with sodium bicarbonate or hot water. Postharvest Biol. Technol. 28, 187–194. 10.1016/S0925-5214(02)00145-X

[ref34] Pavoncello D. Lurie S. Droby S. Porat R. (2001). A hot water treatment induces resistance to *Penicillium digitatum* and promotes the accumulation of heat shock and pathogenesis-related proteins in grapefruit flavedo. Physiol. Plant. 111, 17–22. 10.1034/j.1399-3054.2001.1110103.x

[ref35] Porat R. Daus A. Weiss B. Cohen L. Droby S. (2002). Effects of combining hot water, sodium bicarbonate and biocontrol on postharvest decay of citrus fruit. J. Hortic. Sci. Biotechnol. 77, 441–445. 10.1080/14620316.2002.11511519

[ref36] Quast C. Pruesse E. Yilmaz P. Gerken J. Schweer T. Yarza P.. (2013). The SILVA ribosomal RNA gene database project: improved data processing and web-based tools. Nucleic Acids Res. 41, D590–D596. 10.1093/nar/gks1219, PMID: 23193283 PMC3531112

[ref37] Schloffer K. Linhard D. (2016). “Short time-high temperature hot water shower against Neofabraea rot” in *Ecofruit. 17th International Conference on Organic Fruit-Growing: Proceedings, 15–17 February 2016* (Fördergemeinschaft Ökologischer Obstbau eV (FÖKO)). ed. Kienzle J. (Hohenheim, Germany: FÖKO), 176–179.

[ref38] Shannon P. Markiel A. Ozier O. Baliga N. S. Wang J. T. Ramage D.. (2003). Cytoscape: a software environment for integrated models of biomolecular interaction networks. Genome Res. 13, 2498–2504. 10.1101/gr.1239303, PMID: 14597658 PMC403769

[ref39] Snowdon A. L. (1990). A colour atlas of post-harvest diseases and disorders of fruits and vegetables. Volume 1. UK: Wolfe Scientific Available at: https://www.cabdirect.org/cabdirect/abstract/19901142685 (Accessed July 2, 2019).

[ref40] Spadaro D. Garibaldi A. Gullino M. L. (2004). Control of *Penicillium expansum* and *Botrytis cinerea* on apple combining a biocontrol agent with hot water dipping and acibenzolar-S-methyl, baking soda, or ethanol application. Postharvest Biol. Technol. 33, 141–151. 10.1016/j.postharvbio.2004.02.002

[ref41] Spadaro D. Gullino M. L. (2005). Improving the efficacy of biocontrol agents against soilborne pathogens. Crop Prot. 24, 601–613. 10.1016/j.cropro.2004.11.003

[ref42] Tannous J. Atoui A. El Khoury A. Kantar S. Chdid N. Oswald I. P.. (2015). Development of a real-time PCR assay for *Penicillium expansum* quantification and patulin estimation in apples. Food Microbiol. 50, 28–37. 10.1016/j.fm.2015.03.001, PMID: 25998812

[ref43] Thompson A. K. Prange R. K. Bancroft R. Puttongsiri T. (2018) in Controlled atmosphere storage of fruit and vegetables. eds. Thompson A. K. Prange R. K. Bancroft R. D. Puttongsiri T. (Wallingford: CABI).

[ref44] Trierweiler B. Schirmer H. Tauscher B. (2003). Hot water treatment to control Gloeosporium disease on apples during long-term storage. J. Appl. Bot. 77, 156–159.

[ref45] Vandenkoornhuyse P. Quaiser A. Duhamel M. Le Van A. Dufresne A. (2015). The importance of the microbiome of the plant holobiont. New Phytol. 206, 1196–1206. 10.1111/nph.13312, PMID: 25655016

[ref46] Wszelaki A. L. (2003). Effect of combinations of hot water dips, biological control and controlled atmospheres for control of gray mold on harvested strawberries. Postharvest Biol. Technol. 27, 255–264. 10.1016/S0925-5214(02)00095-9

[ref47] Zhang H. Wang S. Huang X. Dong Y. Zheng X. (2008). Integrated control of postharvest blue mold decay of pears with hot water treatment and *Rhodotorula glutinis*. Postharvest Biol. Technol. 49, 308–313. 10.1016/j.postharvbio.2008.01.004

[ref48] Zong Y. Liu J. Li B. Qin G. Tian S. (2010). Effects of yeast antagonists in combination with hot water treatment on postharvest diseases of tomato fruit. Biol. Control 54, 316–321. 10.1016/j.biocontrol.2010.06.003

