# Supplementary material for: Microbiome Response to Hot Water Treatment and Potential Synergy With Biological Control on Stored Apples
Source: Front Microbiol. 2019 Nov 6;10:2502. doi: 10.3389/fmicb.2019.02502 (PMC6852696; doi:10.3389/fmicb.2019.02502)
Supplement: Supplementary file 1 [file Data_Sheet_1.docx]

***Supplementary Material***


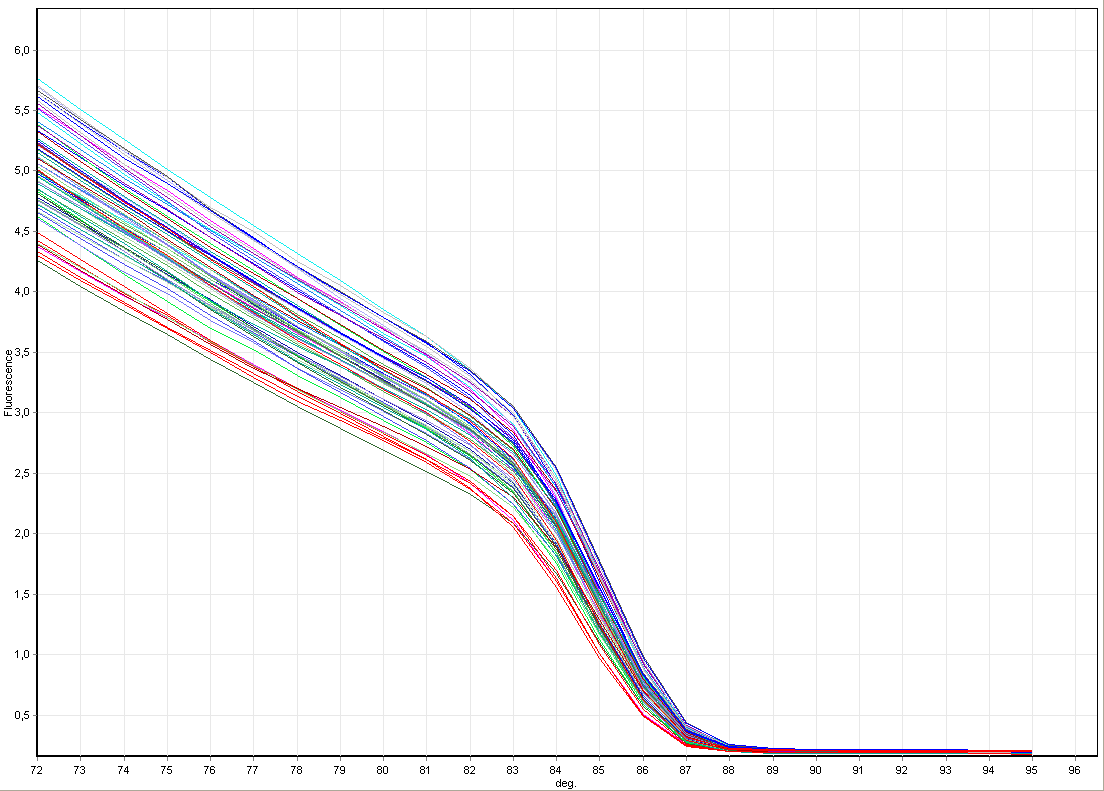


**Supplementary Figure 1:** Analyzation of the melting curve of qPCR samples after amplification. Melting was performed after each run using a heat gradient from 72 to 95°C.

**
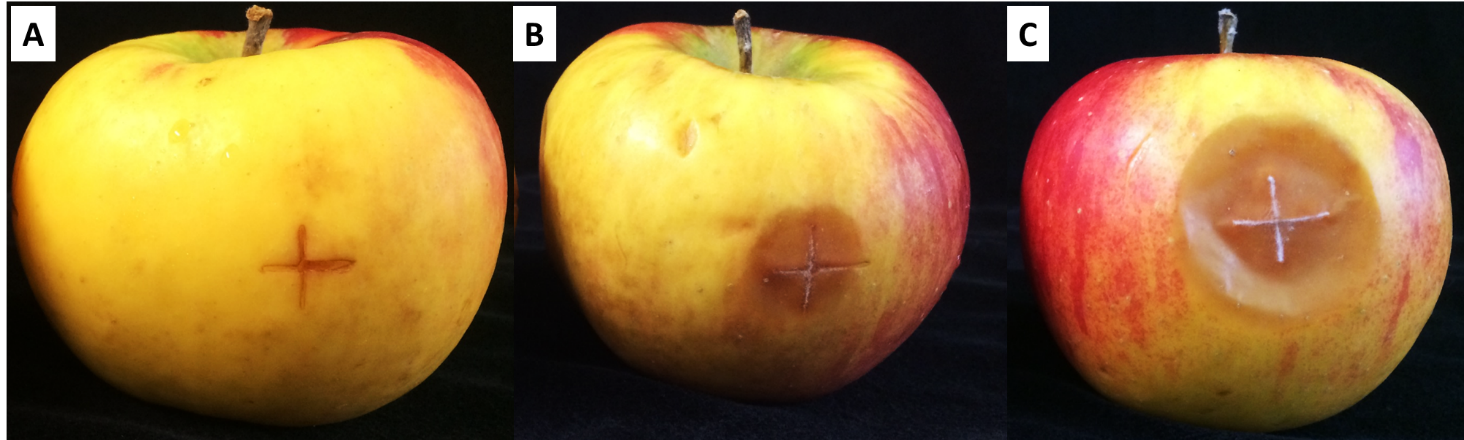
**

**Supplementary Figure 2:** Pathosystem of *P. expansum* on Topaz apples directly (A), one week (B) and three weeks (C) after artificial wounding over the course of small scale storage experiments. Diameters of infected areas were measured after three weeks of storage.

**Supplementary Table 1:** Primers used in the present study.

| **Primer name** | **Primer sequence 5’-3’** | **Reference** |
| --- | --- | --- |
| 515f | GTGYCAGCMGCCGCGGTAA | (Caporaso et al. 2010) |
| 927r | CCGYCAATTYMTTTRAGTTT |  |
| ITS 1f | CTTGGTCATTTAGAGGAAGTAA | (White et al. 1990) |
| ITS 2r | GCTGCGTTCTTCATCGATGC |  |
| Pexp_patF_F | ATGAAATCCTCCCTGTGGGTTAGT | (Tannous et al. 2015) |
| Pexp_patF_R | GAAGGATAATTTCCGGGGTAGTCATT |  |
| NeoF | CTTTCTCCGTTGTCCCATCC | (Cao et al. 2013) |
| NeoR | GAACATTGCGCATCTGGTCC |  |

**Supplementary Table 2:** Bacterial and fungal genera with significantly different abundance in ‘untreated healthy’ and ‘untreated diseased’ apples.

|  | | **untreated healthy** | **untreated diseased** | **FDR_P** |
| --- | --- | --- | --- | --- |
| Bacterial genera | *Sphingomonas* | **18875** | 3009 | 0.00 |
|  | *Pseudomonas* | **4839** | 343 | 0.00 |
|  | *Methylobacterium* | **2945** | 762 | 0.00 |
|  | *Hymenobacter* | **2456** | 92 | 0.00 |
|  | *Massilia* | **1464** | 0 | 0.00 |
|  | *Novosphingobium* | **911** | 187 | 0.00 |
|  | *f_Microbacteriaceae* | **722** | 69 | 0.00 |
|  | *f_Burkholderiaceae* | **704** | 103 | 0.00 |
|  | *Ralstonia* | **621** | 94 | 0.00 |
|  | *Kineococcus* | **523** | 188 | 0.00 |
|  | *Spirosoma* | **504** | 82 | 0.00 |
|  | *Mucilaginibacter* | **390** | 0 | 0.00 |
|  | *Burkholderia* | **363** | 0 | 0.00 |
|  | *Roseomonas* | **323** | 0 | 0.00 |
|  | *Rathayibacter* | **303** | 116 | 0.01 |
|  | *Amnibacterium* | **302** | 0 | 0.00 |
|  | *f_Sphingomonadaceae* | **301** | 99 | 0.01 |
|  | *Geodermatophilus* | **285** | 112 | 0.00 |
|  | *f_Beijerinckiaceae* | **277** | 86 | 0.00 |
|  | *Clostridium sensu stricto 1* | **230** | 0 | 0.00 |
|  | *Nocardioides* | **156** | 0 | 0.00 |
|  | *k_Bacteria* | **145** | 0 | 0.00 |
|  | *Acidiphilium* | **134** | 0 | 0.00 |
|  | *Deinococcus* | **133** | 0 | 0.00 |
|  | *Basidiomycota* | **130** | 0 | 0.00 |
|  | *f_Kineosporiaceae* | **125** | 0 | 0.00 |
|  | *Aureimonas* | **125** | 0 | 0.00 |
|  | *Patulibacter* | **116** | 0 | 0.00 |
|  | *o_Armatimonadales* | **115** | 0 | 0.00 |
|  | *f_Nocardioidaceae* | **109** | 0 | 0.00 |
|  | *Aeromicrobium* | **108** | 0 | 0.01 |
|  | *Bdellovibrio* | **96** | 0 | 0.00 |
|  | *Pedobacter* | **94** | 0 | 0.02 |
|  | *Pajaroellobacter* | **82** | 0 | 0.00 |
|  | *Nakamurella* | **77** | 0 | 0.01 |
|  | *uncultured bacterium* | **74** | 0 | 0.00 |
|  | *uncultured* | **74** | 0 | 0.02 |
|  | *Terriglobus* | **73** | 0 | 0.01 |
|  | *Acinetobacter* | **62** | 0 | 0.01 |
|  | *k_Bacteria* | **62** | 0 | 0.01 |
|  | *Terrisporobacter* | **59** | 0 | 0.00 |
|  | *Belnapia* | **50** | 0 | 0.00 |
|  | *Jatrophihabitans* | **34** | 0 | 0.02 |
|  | *f_Fimbriimonadaceae* | **28** | 0 | 0.02 |
|  | *f_Acetobacteraceae* | **25** | 0 | 0.02 |
| Fungal genera | *Vishniacozyma* | **4810** | 588 | 0.00 |
|  | *Cladosporium* | **3948** | 454 | 0.00 |
|  | *f_Didymellaceae* | **3233** | 574 | 0.00 |
|  | *o_Hypocreales* | **2622** | 382 | 0.01 |
|  | *Acremonium* | **1222** | 0 | 0.00 |
|  | *Mycosphaerella* | **1183** | 121 | 0.00 |
|  | *p_Ascomycota* | **931** | 125 | 0.00 |
|  | *Leptosphaeria* | **438** | 175 | 0.01 |
|  | *k_Fungi* | **419** | 0 | 0.00 |
|  | *o_Hypocreales* | **294** | 0 | 0.00 |
|  | *Filobasidium* | **274** | 39 | 0.00 |
|  | *Alternaria* | **203** | 45 | 0.00 |
|  | *f_Didymellaceae* | **200** | 71 | 0.02 |
|  | *Ramularia* | **196** | 40 | 0.00 |
|  | *p_Basidiomycota* | **180** | 24 | 0.00 |
|  | *unidentified* | **156** | 0 | 0.00 |
|  | *Symmetrospora* | **106** | 15 | 0.00 |
|  | *Uncobasidium* | **68** | 0 | 0.02 |
|  | *Bullera* | **30** | 0 | 0.00 |
|  | *f_Phaeosphaeriaceae* | **23** | 0 | 0.00 |
|  | *Cystobasidium* | **23** | 0 | 0.00 |
|  | *Bensingtonia* | **22** | 0 | 0.00 |
|  | *f_Mycosphaerellaceae* | **19** | 0 | 0.01 |
|  | *f_Cystobasidiaceae* | **15** | 0 | 0.00 |
|  | *f_Sporidiobolaceae* | **11** | 0 | 0.01 |
|  | *Sporobolomyces* | **10** | 0 | 0.01 |
|  | *Aureobasidium* | **9** | 0 | 0.02 |
|  | *Kurtzmanomyces* | **8** | 0 | 0.02 |
|  | *P. expansum* | 284 | **9122** | 0.02 |
|  | *N. alba* | 804 | **8512** | 0.02 |

*Abundance in absolute hits that was significantly higher in the respective apple group, is highlighted in bold. **Significances were calculated by applying non-parametric Kruskal-Wallis/FDR-P (alpha=0.05).

**Supplementary Table 3:** Bacterial and fungal genera with significantly different abundance in ‘HWT’ and ‘untreated healthy’ apples.

|  | **Taxonomy** | **HWT*** | **untreated healthy*** | **FDR_P**** |
| --- | --- | --- | --- | --- |
| Bacterial genera | *Hymenobacter* | **2456** | 801 | 0.02 |
|  | *Rathayibacter* | **303** | 200 | 0.04 |
|  | *Amnibacterium* | **302** | 104 | 0.02 |
|  | *k_Bacteria* | **145** | 30 | 0.03 |
|  | *Basidiomycota* | **130** | 10 | 0.01 |
|  | *f_Solirubrobacteraceae* | **109** | 0 | 0.01 |
|  | *Pedobacter* | **94** | 0 | 0.03 |
|  | *o_Myxococcales* | **74** | 0 | 0.01 |
|  | *f_Beijerinckiaceae* | **74** | 0 | 0.03 |
|  | *k_Bacteria* | **62** | 0 | 0.02 |
|  | *Terrisporobacter* | **59** | 0 | 0.01 |
|  | *Belnapia* | **50** | 0 | 0.01 |
|  | *Jatrophihabitans* | **34** | 0 | 0.03 |
|  | *f_Fimbriimonadaceae* | **28** | 0 | 0.03 |
|  | *Curtobacterium* | 66 | **1268** | 0.01 |
|  | *Rhodococcus* | 0 | **286** | 0.01 |
|  | *Meiothermus* | 0 | **174** | 0.01 |
|  | *Flavisolibacter* | 0 | **132** | 0.01 |
|  | *Marmoricola* | 0 | **107** | 0.02 |
|  | *Turicibacter* | 0 | **55** | 0.02 |
|  | *Gemmata* | 0 | **54** | 0.01 |
|  | *f_Blastocatellaceae* | 0 | **50** | 0.02 |
|  | *Lacibacter* | 0 | **39** | 0.03 |
|  | *f_Nocardioidaceae* | 0 | **37** | 0.03 |
|  | *p_Armatimonadetes* | 0 | **18** | 0.03 |
| Fungal genera | *f_Didymellaceae* | **3233** | 1766 | 0.04 |
|  | *p_Ascomycota* | **931** | 286 | 0.01 |
|  | *k_Fungi* | **419** | 95 | 0.02 |
|  | *o_Hypocreales* | **294** | 0 | 0.00 |
|  | *Filobasidium* | **274** | 154 | 0.04 |
|  | *p_Basidiomycota* | **180** | 39 | 0.01 |
|  | *o_Entylomatales* | **177** | 0 | 0.01 |
|  | *Symmetrospora* | **106** | 41 | 0.02 |
|  | *Uncobasidium* | **68** | 0 | 0.03 |
|  | *Bullera* | **30** | 0 | 0.00 |
|  | *Bensingtonia* | **22** | 0 | 0.00 |
|  | *f_Mycosphaerellaceae* | **19** | 0 | 0.01 |
|  | *o_Capnodiales* | **15** | 0 | 0.01 |
|  | *f_Cystobasidiaceae* | **11** | 0 | 0.01 |
|  | *Kurtzmanomyces* | **8** | 0 | 0.03 |
|  | *Penicillium* | 284 | **1976** | 0.01 |
|  | *f_Nectriaceae* | 0 | **718** | 0.00 |
|  | *Alternaria* | 203 | **571** | 0.01 |
|  | *Cystobasidium* | 23 | **186** | 0.00 |
|  | *Aureobasidium* | 9 | **54** | 0.01 |
|  | *f_Apiosporaceae* | 0 | **18** | 0.01 |
|  | *Leptospora* | 0 | **9** | 0.01 |

*Abundance in absolute hits that was significantly higher in the respective apple group, is highlighted in bold. **Significances were calculated by applying non-parametric Kruskal-Wallis/FDR-P (alpha=0.05).
